# Implementation of a deprescribing guideline by community pharmacists: A focus group study

**DOI:** 10.1016/j.rcsop.2026.100813

**Published:** 2026-06-10

**Authors:** Sanne Bakker, Gert Baas, Marcel L. Bouvy, Petra Denig, Mette Heringa

**Affiliations:** a SIR Institute for Pharmacy Practice and Policy, 2331 JE Leiden, the Netherlands; b Department of Pharmaceutical Sciences, Utrecht University, 3584 CS Utrecht, the Netherlands; c Department of Clinical Pharmacy and Pharmacology, University of Groningen, University Medical Center Groningen, 9700 RB Groningen, the Netherlands

**Keywords:** Deprescribing, Polypharmacy, Older adults, Implementation, Community pharmacy

## Abstract

**Background:**

Polypharmacy is common in older adults and, independent of underlying multimorbidity, is associated with adverse drug events, reduced quality of life, and increased healthcare use. Deprescribing can help address these issues. Pharmacists can identify deprescribing opportunities during clinical medication reviews (CMRs), and guidelines may support this process. However, their impact depends on successful implementation.

**Objectives:**

To identify determinants relevant for the implementation of this deprescribing guideline based on CPs' experiences with its use in clinical practice.

**Methods:**

Online focus groups were held with community pharmacists from the intervention group of a cluster randomized trial evaluating guideline-assisted deprescribing during CMRs in older patients with hyperpolypharmacy (≥10 medications). Pharmacists received training on the guideline and its drug-specific fact sheets. The topic guide was informed by the *Tailored Implementation for Chronic Diseases* (TICD) checklist. Transcripts were deductively coded and analysed thematically.

**Results:**

Nineteen of 24 eligible pharmacists participated in three focus groups. Across seven TICD domains, 26 determinants were identified. There were few barriers related to the guideline itself, with pharmacists appreciating the layered structure and summaries and applicability of recommended interventions. At personal level, their ability to identify deprescribing opportunities and to monitor clinical outcomes was considered relevant. . At patient level, barriers included limited knowledge and ambivalent attitudes. Key influences regarding professional interaction included general practitioners' familiarity with deprescribing, clarity of task allocation, and interprofessional trust. Regarding resources insufficient reimbursement, and lack of integrated decision support were reported as barriers.

**Conclusions:**

Drug-specific fact sheets support deprescribing but do not fully address the complexity of decision-making in practice. Implementation is influenced by clinical uncertainty, experience, interprofessional collaboration, and organisational factors. Strengthening decision support, collaboration, and organisational conditions is essential for sustainable implementation.

## 1 Introduction

Due to the advances in healthcare and the availability of effective medicines, people are living longer than ever before. However, this also led to an increase of medication use, especially in older people. In the Netherlands, 42% of individuals aged 75 and above use five or more medications, which is referred to as polypharmacy.^1^ In frail older patients, polypharmacy is associated with an increased risk of adverse drug reactions and, beyond its relationship with multimorbidity, may independently impair health-related quality of life, particularly when prescribing is inappropriate. Consequently, polypharmacy has been linked to poorer patient outcomes, including reduced quality of life, increased (drug-related) hospitalisations, higher healthcare costs, and increased mortality.^2^, ^3^ Moreover, some preventive medication may no longer provide meaningful benefits for the frail elderly.^4^ A 2017 Dutch study showed that 34.7% of patients aged 65 years and older were prescribed potentially inappropriate medications.^5^ To address this, deprescribing – the planned process of dose reduction or discontinuation of medications that may no longer be beneficial or may cause harm, supervised by a health care professional (HCP) – aims to manage polypharmacy and improve patient outcomes.^6^

Despite its potential, deprescribing is not yet routine practice. Barriers include limited training and communication skills among professionals, poor interprofessional coordination, and insufficient evidence or practical guidance to support decision-making.^6^, ^7^, ^8^ Studies indicate that the existence of a guideline itself may serve as a motivational factor for deprescribing and that the presence of such guidelines can enhance the self-efficacy of HCPs.^10^, ^11^ Guidelines provide structure and legitimacy to the deprescribing process, supporting professionals in making complex clinical decisions and in communicating these with patients and other HCPs. However, awareness of existing deprescribing guidelines is not universal and the use of guidelines does not consistently lead to a significant reduction in medication use.^12^, ^13^ Implementation of guideline recommendations may depend on a variety of other factors, such as interprofessional collaboration and communication and time constraints. This multifactorial nature of implementation is well captured in the TICD checklist by Flottorp et al., which conceptualises implementation determinants across interacting domains, including the guideline itself (its form and content), characteristics of the HCP and patient, the quality of interprofessional collaboration, and features of the wider healthcare system at both local and national levels.^14^, ^15^

In 2020, a multidisciplinary Dutch deprescribing guideline was published, including a general part on the deprescribing process and ten drug specific deprescribing fact sheets.^16^ Community pharmacists (CPs) are expected to be key users of the guideline, as a clinical medication review (CMR) – a recommended approach for deprescribing – is typically led by CPs in the Netherlands. Dutch CPs share responsibility for pharmacotherapy with prescribers and CMRs provide an opportunity to identify deprescribing opportunities and formulate recommendations for treatment modifications in collaboration with general practitioners (GPs) and other prescribers. While barriers and facilitators related to deprescribing in general have been studied before, determinants relevant to the implementation and practical use of a newly developed multidisciplinary deprescribing guideline in routine community pharmacy practice remain underexplored. To support the implementation of the guideline, training is offered to CPs to make them aware of the recommendations and how to apply them in practice. This study aims to identify determinants relevant for the implementation of this deprescribing guideline based on CPs' experiences with its use in clinical practice.

## 2 Methods

### 2.1 Study design

This qualitative study was reported in accordance with the Consolidated Criteria for Reporting Qualitative Research (COREQ).^17^ A qualitative sub-study was conducted within a cluster randomized trial (initiated in October 2022) that evaluated the effect of CMRs with a focus on deprescribing in older patients with hyperpolypharmacy (≥ 10 medicines) using a multidose drug dispensing (MDD) system. In this qualitative sub-study, online focus group sessions with 6–10 participants were conducted with CPs from the intervention group of the trial. Focus groups were selected because the interactive group setting was expected to stimulate reflection, comparison of experiences, and discussion.

### 2.2 Deprescribing guideline

The Dutch guideline addresses both barriers and facilitators to deprescribing, as well as the evidence on its effects and the identification of suitable patients, timing, and tools.^16^ At the time of the trial, the guideline included ten drug specific deprescribing fact sheets each focusing on a specific drug class with somatic (e.g., proton pump inhibitors) or preventive indications (e.g., anti-hypertensive drugs). Each fact sheet follows a standardized layered format comprising: i) a summary card with recommendations for deprescribing, ii) an explanatory section detailing these recommendations, iii) a stepwise tapering protocol, iv) an overview of the potential benefits and risks of deprescribing, v) a summary of available evidence for (frail) older patients, and vi) detailed footnotes including references to the relevant literature.

### 2.3 Setting and participants

CPs in the intervention group (*n* = 29) of the trial were expected to join a one-day training (*n* = 28 participated) provided by expert trainers. The training was based on previous training programs,^18^ covering the main barriers identified in previous research and the topics covered in the guideline: 1) the organization of CMR focusing on deprescribing, 2) communication skills to support deprescribing consultations, and 3) the application of drug specific deprescribing fact sheets in practice. After the training, the CPs applied the guideline and drug specific deprescribing fact sheets in CMRs for patients included in the trial.

For this qualitative study, CPs were eligible if they had conducted at least one CMR within the trial, ensuring they had experience with deprescribing and application of the guideline and drug-specific fact sheets, enabling them to provide relevant reflections on implementation experiences. Eligible CPs (*n* = 24) were invited by e-mail to participate in focus group sessions. Reminders were sent when needed, and if no response was received, follow-up contact was made by telephone.

### 2.4 Topic guide

A topic guide was developed based on themes from Flottorp's Tailored Implementation for Chronic Diseases (TICD) checklist,^14^ which is used to identify determinants of HCP practice that influence implementation. The 7 domains of the checklist were condensed to five topics in the topic guide: i) drug specific deprescribing fact sheets, ii) individual factors of HCPs, iii) patient factors, iv) professional collaboration, and v) organization and embedding (including relevant aspects from the TICD domains ‘Incentives and resources’, ‘Capacity for organisational change’ and ‘Social, political and legal factors’), see Appendix A. After the first focus group, the topic guide was reevaluated by the researchers and moderator. The topics covered in the guide remained consistent but the guide was refined to address feasibility in domain ‘Guideline factors’ and implementation of deprescribing recommendations in domain ‘Individual HCP factors’ more explicitly.

### 2.5 Data collection

To address challenges faced by physical sessions, such as high dropout rates and high time investment,^19^ online focus group sessions were conducted using Microsoft Teams. The spoken language was Dutch. One experienced moderator (PD) and two co-moderators (GB and LW) led the focus group sessions. The moderator ensured adherence to research objectives, equal participation, and posed follow-up questions. The co-moderators managed technology, took notes, observed behaviour and occasionally posed clarifying questions.

### 2.6 Data analysis

The focus group recordings were transcribed using Amberscript, which was reviewed for errors and corrected by two researchers (GB and LW). Coding was deductively guided by Flottorp's TICD checklist while allowing refinement and interpretation of themes within domains during the iterative analytical process.^14^ The transcripts were independently coded using NVivo by two researchers, a community pharmacist / Ph.D. candidate and a Master's Pharmacy student (GB and LW). Coding was carried out by reading the transcripts and linking codes to the relevant (sub)domains within the framework. Subsequently, deductive thematic analysis was performed within the TICD framework domains by two researchers / community pharmacists (GB and SB). A comparative analysis of the codes was conducted by the two researchers to identify similarities and differences with the goal of achieving consensus. In the end, the identified determinants were discussed and agreed upon with the full research team.

### 2.7 Ethics and confidentiality

The Medical Ethical Review Committee NedMec (METC NedMec) concluded that this study (21/627) fell outside the scope of the Medical Research Involving Human Subjects Act (WMO). The Utrecht Pharmacy Practice Network for Education and Research (UPPER) Institutional Review Board approved the study protocol (UPF2303) as an addition to the original trial protocol (UPF2211). The participating CPs provided written consent for study participation. and gave verbal consent for audio recording at the start of the focus group discussions. Details in focus groups transcripts that could potentially identify the participants or specific locations were anonymized.

## 3 Results

Of the 24 invited CPs who met the inclusion criteria, 19 took part in three online focus group sessions (October–November 2023), each lasting approximately 120 min. Participant characteristics are shown in Table 1, and detailed information for individual participants is provided in Appendix B. Reasons for not participating in the focus groups were lack of time, no response and personal circumstances, such as pregnancy leave.

**Table 1:** Distribution of participants per session with additional demographic information.

Characteristic	Session 1	Session 2	Session 3
Number of participants	7	6	6
Female	7	4	5
Practice in urban vs rural settings	4 vs 3	3 vs 3	6 vs 0
Number of performed clinical medication reviews during the trial (range)	3–18	4–11	1–10

Within the seven TICD domains, 26 determinants were identified (Table 2). In the third focus group session, no new determinants appeared. No additional overarching themes outside the TICD framework emerged during analysis.

**Table 2:** Overview of identified determinants for practice categorized in the (sub)domains of the TICD checklist.^14^

TICD Domain		TICD Subdomain	Identified determinants	Enabler (+), barrier (−), mixed (=)
1.	Guideline factors	Recommendation	Layered structure of the deprescribing fact sheets	+
			Concise synthesis of evidence	+
		Recommended clinical intervention	Applicability to patients with high medication burden	+
			Use of MDD-systems considered as an advantage for implementing deprescribing	+
			Role of informal caregivers in deprescribing decisions	=
			Need for practical risk assessment tools	−
		Recommended behaviour	Importance of measurable clinical effects to monitor deprescribing	=
2.	Individual HCP factors	Knowledge and skills	Consultation skills for initiating deprescribing	=
			Clinical experience in identifying deprescribing opportunities	+
			Lack of monitoring limits learning from intervention outcomes	−
		Cognitions (including attitudes)	Fluctuating motivation to pursue deprescribing	−
			Fear and hesitation related to professional responsibility	−
		Professional behaviour	Limited capacity to plan and sustain follow-up	−
3.	Patient factors		Emotional reluctance and ambivalent motivation towards deprescribing	=
			Limited medication knowledge hindering shared decision making	−
			High trust in physicians' authority	=
			Complexity due to multimorbidity	−
4.	Professional interactions		Limited GP awareness of guideline recommendations and willingness towards deprescribing	−
			Unclear allocation of tasks and responsibilities	−
			Development of trust between CPs and GPs	+
5.	Incentives and resources		Need for additional drug-specific deprescribing fact sheets	−
			Need for integrated clinical decision-support	−
			Variation and insufficient reimbursement for deprescribing	−
			Need for information on patient level language	−
6.	Capacity for organisational change		Preference for starting with low-complexity cases	+
7.	Social, political and legal factors		Lack of policy recognition and inclusion of deprescribing in contracts	−

Abbreviations: TICD = tailored implementation for chronic diseases; MDD = multidose drug dispensing; HCP = healthcare professional; CP = community pharmacist; GP = general practitioner.

### 3.1 Guideline factors

#### 3.1.1 Recommendation

The drug specific ***deprescribing fact sheets*** were appreciated for compiling all information into one ***layered document***, which made them easy to use in daily practice. Most CPs valued the summaries and pros/cons which provided clear guidance while supporting individual choice, whereas footnotes were usually appreciated for completeness providing ***concise synthesis of evidence***.

*“For me, the core information is on the first few pages [summary, concrete recommendations and pros and cons list], and I have used that. I haven't really delved into the footnotes, but it's nice that it's comprehensive.”* P19.

However, one CP indicated that for certain medication groups, more detailed evidence or information on the strength of evidence was desired. In particular, the available information was sometimes perceived as insufficient to justify deprescribing for specific drug classes, such as anticoagulants.

“*If my cardiologists asks, ‘Gee, you discontinued that acetylsalicylic acid, please provide the evidence.’ Yes I wonder if the drug specific deprescribing fact sheets provides sufficient support to explain that.”* P10.

#### 3.1.2 Recommended clinical intervention

Discussions about implementability of the recommendations focused on four determinants related to the guideline's target group: older patients (≥ 75) with (hyper)polypharmacy, who may use an MDD or have an informal caregiver.

According to several CPs, recommendations for deprescribing are easier to implement for ***patients with a high medication burden***, as this often means there is more unnecessary medication in the regimen.

*“You do notice that the more medications people use, the more there is that is actually no longer necessary.”* P4.

***Use of an MDD-system*** was considered an ***advantage for implementing deprescribing*** recommendations as it facilitates tapering, prevents unnecessary medication stockpiling and simplifies regimen changes for patients, by reducing the need to remember complex dosing schedules. Furthermore, it was noted that medications in the MDD are rarely reviewed, leading to the prolonged use of medications that could actually be deprescribed.

*“The use of an MDD is convenient, allowing you to clearly implement what you have agreed upon, especially during tapering. Having a well-defined schedule can greatly assist patients in that regard.”* P5.

Perspectives on ***informal caregivers' role*** were mixed: they could help recall patient's concerns and keep discussions focused, but problems arose when they dominated conversations, diverge form the patient's perspective, or assume control over decision-making.

*“When the partner starts speaking for the person you are assessing, it is sometimes helpful because they often know the details well. However, it can also be a bit annoying, preventing the other person from expressing themselves.”* P19.

Regarding ***risk assessment tools**, instruments* like the International Prostate Symptom Score were considered helpful for implementing deprescribing. In contrast, other tools were perceived as complex due to the extensive data entry and difficulties in communicating results to patients, highlighting the need for more practical tools.

#### 3.1.3 Recommended behaviour

To effectively implement deprescribing recommendations, CPs preferred situations in which the ***clinical effects*** of an intervention were ***measurable*** (e.g., blood pressure).

*“Consider antihypertensives; you can monitor blood pressure. But especially with interventions where you can't monitor it so effectively, where you basically must believe it, based on the evidence. Yes, that, I find challenging*.” P10.

### 3.2 Individual HCP factors

#### 3.2.1 Knowledge and skills

CPs generally reported sufficient ***consultation skills*** though some CPs reported occasional challenges in initiating deprescribing discussions.

*“To initiate discussions about discontinuation, they [patients] are not all that receptive, so I found that challenging. And you really have to come up with strong arguments.”* P18.

CPs generally considered their ***clinical experience and expertise*** sufficient to identify deprescribing opportunities*,* which sometimes rendered the deprescribing fact sheets unnecessary, as the information was already familiar to them. However, confidence appeared stronger for familiar medication classes and situations in which outcomes could be monitored more easily.

*“As a pharmacist, you have extensive knowledge, and there's also a bit of clinical ability. You understand the drawbacks of products and how these drawbacks may escalate with age.”* P10.

CPs acknowledged ***lacking monitoring*** deprescribing outcomes, where they could learn from ***the outcomes of their recommended interventions***.

*“I often find myself lacking, and I think it's a matter of experience, in not consistently receiving feedback on the outcomes of my interventions. So, often, you don't know the impact of your intervention on the patient.”* P4.

#### 3.2.2 Cognitions (including attitudes)

The ***motivation to pursue deprescribing*** may diminish after unsuccessful attempts despite considerable effort, or when the patient's history indicates that deprescribing is not feasible.

*“You want to make an effort, but it simply didn't work out. The man still has twelve medications, and you can't help but feel quite disappointed. Then you think: why are you doing this? In terms of results, it's yielded nothing.”* P18.

Concerning ***professional responsibility***, participants mainly expressed fear of tapering without sufficient experience or evidence, and the hesitation to assume responsibility for the decision. One participant mentioned that CPs may need to rely on their own clinical reasoning.

*“Do we dare to take the responsibility to make such a choice? Because we [pharmacists] are very accustomed to acting based on certainty. But I think it's admirable if we also dare to act based on a bit of clinical reasoning.”* P11.

#### 3.2.3 Professional behaviour

CPs emphasized the ***limited capacity to plan and sustain follow-up,*** which challenges long-term adherence. Overall, proper follow-up was challenging due to the lengthy nature of the process, requiring careful planning and organization.

*“When I consider the amount of time I'm currently investing in these CMRs, it's truly enormous. Honestly, I find it incredibly enjoyable and interesting, but I won't be able to sustain this pace.”* P4.

### 3.3 Patient factors

Concerning ***emotional reluctance and ambivalent motivation,*** CPs observed varying patient willingness to deprescribe. Although some patients wished to reduce medication, particularly when side effects occurred, many preferred to continue their current medication regimen. Even when patients initially agreed, fear and apprehension often impeded implementation.

*“They are highly motivated to deprescribe, and the moment you give them the green light for it, they suddenly find it quite scary not to take the medication.”* P2.

CPs noted that implementing ***shared decision-making*** was challenging because patients often lacked a clear ***understanding of purpose of the medications*** they use. CPs perceived this population as more deferential and less able to make informed choices.

*“This population seems to have a bit of uncertainty about the purposes of their medications and has somewhat let go of understanding it all. That, in itself, makes it challenging to engage in effective shared decision-making.”* P8.

According to CPs, patients tend to adhere closely to the originally prescribed regimen, with a ***high level of trust in the physician***. This strong reliance on physician authority was perceived to further limit patients' active involvement in deprescribing decisions.

*“You really notice that in this case, it's a group that still strongly adheres to the idea of, ‘Well, the doctor prescribed it, so I just trust that blindly.”* P3.

One mentioned barrier for implementing deprescribing in this specific patient population, was ***complexity due to multimorbidity***.

*“It is also difficult to see a connection over time in complaints or side effects in this population, because they've had these complaints for so long, that it is difficult to see that connection [with medication] sometimes.”* P9*.*

### 3.4 Professional interactions

Concerning GP perceptions, CPs noted ***limited awareness of guideline recommendations*** and the drug-specific fact sheets. Furthermore, GPs and practice nurses were perceived as more focused on initiating medication, with limited awareness of discontinuation as treatment option, resulting in limited willingness and confidence to act.

*“I do notice that it [the deprescribing fact sheets] is still very unfamiliar for GPs, and as a result, the willingness to work with it is not always there yet.”* P4.

Another frequently discussed theme was the ***allocation of tasks and responsibilities**,* when medication was initiated by a specialist. CPs mentioned that GPs sometimes hesitated to modify specialist medications due to this ambiguity, and that the CPs themselves lack a clear point of contact in such situations.

*“I observe a growing trend of, ‘No, this is my [GP] domain, and that's the hospital's domain. And then the hospital says, ‘No, that's no longer under our control, the GP has to handle that now.’ It becomes a game of back-and-forth, and you find yourself [as a pharmacist] caught in the middle.”* P2.

CPs emphasized that a clear division of roles and tasks among CPs, GPs, and practice support staff is crucial. Involving practice support staff in the initiation, planning, and long-term monitoring turned out to be a significant facilitator. However, it remained essential that the GPs provide critical input in this collaborative effort.

*“I believe it is crucial to involve the practice support staff in this process because they ideally see these patients three-monthly. This is where deprescribing should be more prominently addressed.”* P15.

Concerning ***the development of trust between CPs and GPs**,* it was highlighted that many physicians were receptive to deprescribing and express confidence in CPs, but that it takes time to build this confidence. Long-term collaboration and mutual trust were therefore perceived as important facilitators for implementation.

*“I also think that it really matters that there is that trust in the pharmacy and I think that it is also part of the process that simply has to grow over the years after they have seen what we [pharmacists] can do.”* P8.

### 3.5 Incentives and resources

CPs would appreciate ***additional drug specific fact sheets***, for example for psychotropic drugs or inhalation medication used by asthma or COPD.

*“I briefly checked which ones I was missing, and among them were asthma/COPD medications, like stopping inhalation corticosteroids. Someone who uses salbutamol chronically, I'm trying to reduce that too.”* P19.

There was also a desire for integration of ***clinical decision-support*** in information systems to assist selection, scheduling, follow-up, decision support, documentation, and direct linkage to reimbursement.

*“Ideally, you would want to use a clinical decision-support tool that is fully integrated within your pharmacy information system, so that all steps of the process are connected and can be managed in one place.”* P17.

***Reimbursement*** for deprescribing consultations varied between insurers. As a result, some CPs prioritized patients from insurers with higher fees. Furthermore, the current reimbursement model was perceived as unsupportive of deprescribing, as CPs are primarily reimbursed for dispensing, leading to income loss that is not offset by a deprescribing fee.

*“One health insurance company has set a reasonable reimbursement for it this year, which at least covers a portion of the costs. However, it hasn't always been the case. You can't carry out this task for 20 euros. As a pharmacist, you want to do it, and you will do it. But the motivation to conduct, figuratively speaking, 100 reviews for 20 euros, that simply isn't feasible.”* P15.

There was an expressed need for an ***informational leaflet*** accompanying each drug-specific deprescribing fact sheet that is on a ***patient level language***. This leaflet should outline the pros and cons of continued use and deprescribing.

*“It is essential to have a brief section for the patient clearly stating: Why are we doing this? So, for each deprescribing fact sheet, explain in layman's terms what the pros and cons are for them.”* P4.

### 3.6 Capacity for organisational change

The preference was noted to initiate deprescribing with ***low complexity cases***, gradually progressing to more complex scenarios. This approach aimed to assist a larger number of individuals in the short term.

*“I'd rather start with the low-hanging fruit, the [proton pump inhibitors] PPIs and such. Then, each year, I can delve a bit deeper. Because we have to start somewhere, and maybe we should start with the very straightforward things.”* P4.

### 3.7 Social, political and legal factors

CPs mentioned a ***lack of policy recognition and inclusion of deprescribing in contracts***. Regarding contracting, there was a suggestion for health insurers to acknowledge the value of deprescribing and explicitly incorporate it into agreements, potentially with a requirement for a specified number of deprescribing focused CMRs per year.

*“We had an obligation to conduct a certain number of reviews per year. It wasn't ideal, of course, but it ensured that you made time. Now it's a bit more optional, and if you have other priorities that are less flexible, it tends to get pushed back.”* P19.

## 4 Discussion

This study showed that the fact sheets of the deprescribing guideline were considered helpful but not sufficient to fully support CPs to implement deprescribing in practice. Overall, 26 determinants were identified, primarily within the domains of *guideline factors, individual healthcare professional (HCP) factors, patient factors, and professional interactions*. CPs expressed a need for more systematic monitoring and feedback on deprescribing outcomes to build experience. It was acknowledged that deprescribing requires clinical reasoning beyond following guideline recommendations and taking responsibility for doing so. No mention was made of the more general guideline recommendation to support the implementation of deprescribing although several barriers and some enablers at interprofessional, organisational and policy level were mentioned. Known barriers related to patient factors, professional interactions and resources were not resolved yet.

With regard to *guideline factors*, the fact sheets accompanying the guideline were generally perceived as clear, structured, and easy to use. In particular, the layered format, concise summaries, and explicit presentation of benefits and risks can be expected to facilitate the uptake of guideline, consistent with previous research highlighting the importance of adequate evidence synthesis as well as clear messages and format.^15^ Implementation feasibility was considered good for patients with hyperpolypharmacy using an MDD-system, as it facilitates structured dose tapering and medication discontinuation. At the same time, participants indicated that the guideline provided insufficient support in situations characterised by clinical uncertainty or limited evidence, particularly in complex patients or when outcomes are not easily measurable. While the guideline outlines *what* steps should be taken, it offers less guidance on *how* to apply clinical reasoning and communicate with patients about risks and benefits in such contexts. This gap reflects broader challenges described in deprescribing literature, where lack of practical guidance and evidence contributes to implementation barriers.^7^, ^8^, ^9^, ^20^, ^21^ Strengthening the guideline with additional decision-support tools, more explicit handling of uncertainty, and case-based examples may therefore enhance its implementation in daily practice.

At the level of *individual HCP factors*, confidence and experience emerged as key determinants of implementation. CPs reported greater confidence when deprescribing outcomes could be objectively monitored, which is in line with the guideline's recommendation to regularly evaluate ongoing medication use. Similar findings have been reported previously, where measurable outcomes support both professional confidence and patient engagement in deprescribing processes.^22^ However, uncertainty, limited insight in outcomes, and concerns about professional responsibility remained important barriers, as also described in earlier studies among pharmacists and physicians.^7^, ^20^, ^21^, ^23^ Although the guideline promotes a systematic approach, it provides limited support for efficient planning or developing competence over time. CPs tended to start with low-complexity cases, reflecting a pragmatic learning strategy also observed in other studies on clinical decision-making.^23^ In addition, starting with medications for which deprescribing effects can be monitored or measured relatively easily may help CPs gain practical experience and build confidence in deprescribing conversations and clinical decision-making. These findings suggest that implementation could be strengthened by embedding learning trajectories, facilitating structured feedback on outcomes, and further developing educational initiatives focused on both clinical reasoning and communication skills, in line with the guideline's broader recommendations on training.

*Patient-related* determinants were primarily linked to shared decision-making, attitudes towards medication, and willingness to deprescribe. The guideline explicitly emphasises the importance of incorporating patient perspectives, gives suggestions on how to engage patients and their relatives in decision-making, and prioritising deprescribing actions collaboratively. Nevertheless, CPs reported that patient reluctance, limited medication knowledge, and strong trust in physicians often complicated these processes. These findings are consistent with previous research showing that patients may be ambivalent about deprescribing despite recognising potential benefits.^9^, ^20^, ^21^ This suggests that, while the guideline conceptually supports patient-centred care, additional practical tools are needed to operationalise this in routine consultations. Enhancing the use of patient-oriented materials, decision aids, and communication strategies may help bridge this gap and facilitate meaningful shared decision-making.^24^

*Professional interactions*, particularly between CPs and GPs, were identified as a central determinant of successful implementation. Trust, clear role allocation, and shared responsibility were essential for effective collaboration, which is consistent with the guideline's recommendations for making regional collaboration agreements and supporting alignment between prescribers. These findings are in line with earlier studies demonstrating that interprofessional collaboration is crucial for deprescribing in primary care.^7^, ^25^ At the same time, CPs reported that GPs were often unfamiliar with the guideline and its supporting materials, which limited their engagement in deprescribing decisions. Hesitancy was particularly evident when medications had been initiated by specialists, reflecting unclear boundaries of responsibility, also described in previous research.^20^, ^21^, ^25^, ^26^ While increasing awareness of the guideline among GPs appears necessary, evidence suggests that awareness alone is insufficient to change practice.27., 28 Rather, implementation requires a combination of strategies, including multidisciplinary collaboration, clear task allocation, and the gradual development of trust, as also observed in integrated care models.^29^ Facilitating regular pharmacotherapeutic consultations and actively involving all stakeholders in training may therefore support more consistent implementation.

Other domains, including *incentives and resources, organisational capacity, and broader social, political, and legal factors*, highlighted systemic influences on implementation. CPs reported that the current reimbursement framework disincentivizes deprescribing, as reimbursement is predominantly tied to medication dispensing rather than to the provision of time-intensive patient support required for medication discontinuation. Similar barriers have been reported in other healthcare systems, where lack of appropriate incentives limits the uptake of deprescribing practices.^21^ In addition, organisational constraints such as limited time, insufficient integration of decision-support tools, and lack of structural embedding in workflows further hindered implementation. At a broader level, professional boundaries, legal considerations, and existing prescribing-focused quality indicators were perceived as disincentives for deprescribing. Although the guideline acknowledges these factors and calls for alignment at system level, too little progress has been made. National coordinated efforts are needed across policy, organisational, and technological domains to support the integration of deprescribing into routine care. This may include reimbursement models that better reflect the time-intensive nature of deprescribing activities, integration of deprescribing decision-support into pharmacy information systems, and clearer organisational agreements regarding follow-up and task allocation between pharmacists, GPs, and practice support staff.

### 4.1 Strengths and limitations

A strength of this study is that participating CPs had conducted CMRs in which they applied the guideline in practice, providing real-world insights. In addition, the use of the TICD framework to structure the focus groups and the analysis enabled us to link identified determinants to relevant implementation domains. Three focus groups were held with sufficient participants, resulting in saturation in the last group session. Analysis was conducted by two researchers reaching consensus with only minor adjustments for identifying and classifying the determinants.

Inviting only CPs involved in the trial who performed CMRs, however, can also be considered a limitation. Although this enabled reflections based on real-world experience with the guideline, participants may have been more motivated and confident regarding deprescribing than CPs on average. Pharmacists who had not conducted any CMRs may have experienced additional implementation barriers that were not captured in this study. Of note, the majority of the participants were female, which reflects the current population of CPs in the Netherlands. Furthermore, only CPs were included, and perspectives of GPs, support staff and patients were not directly assessed. While these other perspectives have been explored in previous research on deprescribing, future studies should specifically examine other stakeholders' awareness, perceived usability and application of the guideline to confirm the identified determinants and generate additional insights for implementation. Lastly, formal member checking and negative case analysis were not performed.

## 5 Conclusion

This study shows that the drug-specific fact sheets provided useful support for CPs but these fact sheets in combination with the general guideline recommendations were not sufficient to address all barriers to fully implement deprescribing in daily practice. Deprescribing requires clinical reasoning and proactive planning beyond guideline recommendations, and CPs expressed a need for more experience and feedback to support decision-making under uncertainty. Optimisation requires additional practical training and decision support for CPs, as well as stronger collaboration with GPs, increased GP awareness, clear task allocation and regular feedback on outcomes. Finally, adequate organisational support and reimbursement are essential to embed the use of the guideline and fact sheets sustainably in clinical practice.

## CRediT authorship contribution statement

**Sanne Bakker:** Writing – review & editing, Writing – original draft, Formal analysis, Data curation, Conceptualization. **Gert Baas:** Writing – review & editing, Writing – original draft, Methodology, Formal analysis, Data curation, Conceptualization. **Marcel L. Bouvy:** Writing – review & editing, Supervision, Conceptualization. **Petra Denig:** Writing – review & editing, Supervision, Methodology, Investigation, Conceptualization. **Mette Heringa:** Writing – review & editing, Supervision, Project administration, Methodology, Investigation, Funding acquisition, Formal analysis, Conceptualization.

## Disclosure statement

All authors declare that they have no conflicts of interest to disclose.

## Declaration of generative AI and AI-assisted technologies in the writing process

During the preparation of this work, the author(s) used ChatGPT to refine the wording of the abstract and to improve clarity in selected sections of the text. After using this tool, the author(s) reviewed and edited the content as needed and take(s) full responsibility for the content of the published article.

## Funding

The Dutch organization ZonMw has funded the study (project number 10140021910504), as part of the program ‘Goed Gebruik Geneesmiddelen’ and has no other role than funder. ZonMw is the Dutch national organization for health research and healthcare innovation.

## Declaration of competing interest

The authors declare that they have no competing interests.
