# Supplementary material for: Implementation of a deprescribing guideline by community pharmacists: A focus group study
Source: Explor Res Clin Soc Pharm. 2026 Jun 10;23:100813. doi: 10.1016/j.rcsop.2026.100813 (PMC13285370; doi:10.1016/j.rcsop.2026.100813)
Supplement: Supplementary file 1 — Appendix A [file mmc1.docx]

**Appendix A.** Topic guide for the focus groups with pharmacists, translated to English

**Research question:** What is the usefulness of the guideline ‘Deprescribing’ in the context of a clinical medication review, according to community pharmacists?

Start the conversation by welcoming the participants and explaining the purpose of the focus group discussion: gaining insight into the experiences of healthcare professionals regarding deprescribing in the context of medication reviews and the usability of the tools provided in the guideline deprescribing. You have participated as a healthcare professional in the study on deprescribing medication in older adults using a multidose drug dispensing system, and have conducted medication reviews. The discussion will last a maximum of 120 minutes, including a break. At certain moments, we may use the word cloud or the whiteboard. Please keep your microphone and camera turned on. To process this session, it is necessary to record it. Once the recording starts, please confirm that you agree. After that we will do a brief round of introductions. [start recording] Let everyone agree with recording the session. Brief introduction per participant (Name/ Pharmacy/ Location).

**Domain 1:** Drug specific deprescribing fact sheets (Flottorp domain guideline factors)

Action: Display a fact sheet in Teams to identify the five categories it contains, with the aim of enabling more specificity in later discussions.

*Ask a general question about the experience with and the use of the fact sheets.*

- What is your experience with using the fact sheets in the context of medication reviews, with a focus on deprescribing?
- How have you used the fact sheets?
  - Which sections?
    1. Summary of recommendations
    2. Detailed recommendations
    3. Pro’s and con’s
    4. Evidence on deprescribing
    5. Footnotes
  - Additionally, at what stage in the medication review process were they used: communication with the patient, the physician or in drawing up a treatment plan?
- What is your opinion about the content and structure of the fact sheets?
  - *Clarity & structure (al sections)*
  - *Feasibility of recommendations (sections 1 &2)*
  - *Quality of evidence (sections 4&5)*
  - *Alignment with other guidelines/sources (general)*
  - *What could be improved?*
  - *What additional needs do you have?*
- To what extent have you used the generic deprescribing guideline?

**Domain 2:** Individual factors healthcare professionals (Flottorp domain Individual health professional factors)

We would now like to discuss your own knowledge and skills in performing medication reviews focused on deprescribing.

- Do you feel you possess sufficient competencies to perform these tasks?
  In which areas would you like to further develop yourself?
  - *Patient-centred focus (treatment goals, current health status, life expectancy, patient perceptions)*
  - *Consultation skills: ‘shared decision-making’ and ‘motivational interviewing’*
  - *How did you feel about implementing, for example, deprescribing actions (e.g. challenging, difficult with specialist-prescribed medication, dependent on the medication group)?*

**Domain 3:** Patient factors (Flottorp domain Patient factors)

Welcome back. We would now like to discuss the patients for whom you conducted a medication review focused on deprescribing. In the polypharmacy guideline, it is recommended to proactively conduct CMRs in older adults (≥75 years) and in cases of hyperpolypharmacy. In this case, you conducted medication reviews for this target group, specifically those also using medication supplied via multidose drug dispensing systems.

- Were there specific characteristics of this target group that made applying the guideline more difficult or easier?

Action: Initiate a word cloud in Teams poll:

- - *Frailty*
  - *Multidose drug dispensing (Baxter)*
  - *Hyperpolypharmacy*
  - *Patient – healthcare professional relationship*
  - *Patient perception regarding deprescribing*
  - *Engagement in medication use*
  - *Role of informal caregivers*

**Domain 4:** Professional collaboration (Flottorp domain Professional interactions)

The guideline also addresses aspects of collaboration between healthcare professionals that can facilitate deprescribing.

- Could you describe which aspects of collaboration with the general practitioner(s) were important or decisive in deprescribing? What facilitated this and what created challenges?
  - *Attitude of the collaborating GP regarding:*
    - *Whether or not to deprescribe specific medication groups*
    - *Medication initiated by another prescriber*
  - *Role and task distribution*
    - *Monitoring the patient/outcomes*

**Domain 5:** Organisation and embedding (Flottorp domains Incentives and resources, Capacity for organisational change and Social, political and legal factors)

You have conducted MBOs focused on deprescribing for a number of patients in this specific target group.

- What is needed to make deprescribing a structural part of care for more or all patients? What facilitates this?
  - *Multidose drug dispensing process in pharmacy*
  - *Reimbursement for provided care*
  - *Funding model multidose drug dispensing system*
  - *Workload/staffing levels*

Closing question: Are there any other aspects we have not yest addressed that you would like to discuss?
