# Supplementary material for: Implementation of a deprescribing guideline by community pharmacists: A focus group study
Source: Explor Res Clin Soc Pharm. 2026 Jun 10;23:100813. doi: 10.1016/j.rcsop.2026.100813 (PMC13285370; doi:10.1016/j.rcsop.2026.100813)
Supplement: Supplementary file 2 — Appendix B [file mmc2.docx]

**Appendix B.** Distribution of individual participants with participant code, session number, gender, location of the working pharmacy and number of inclusions.

| Participant code | Session | Sex | Practice in urban vs rural settings | Number of performed clinical medication reviews during the trial |
| --- | --- | --- | --- | --- |
| P1 | 1 | Female | City | 8 |
| P2 | 1 | Female | Countryside | 11 |
| P3 | 1 | Female | Countryside | 18 |
| P4 | 1 | Female | City | 3 |
| P5 | 1 | Female | City | 6 |
| P6 | 1 | Female | Countryside | 11 |
| P7 | 1 | Female | City | 6 |
| P8 | 2 | Female | Countryside | 4 |
| P9 | 2 | Female | City | 11 |
| P10 | 2 | Male | Countryside | 6 |
| P11 | 2 | Female | City | 5 |
| P12 | 2 | Female | Countryside | 6 |
| P13 | 2 | Male | City | 9 |
| P14 | 3 | Female | City | 1 |
| P15 | 3 | Female | City | 6 |
| P16 | 3 | Female | City | 6 |
| P17 | 3 | Female | City | 9 |
| P18 | 3 | Female | City | 4 |
| P19 | 3 | Male | City | 10 |
